# Enjoyment, boredom, and perceived effectiveness of learners in language MOOCs: the mediating effect of self-regulated learning

**DOI:** 10.3389/fpsyg.2023.1145773

**Published:** 2023-06-15

**Authors:** Rong Luo, Yijin Wang

**Affiliations:** ^1^School of International Studies, Hangzhou Normal University, Hangzhou, China; ^2^Jing Hengyi School of Education, Hangzhou Normal University, Hangzhou, China

**Keywords:** LMOOCs, self-regulated learning, foreign language enjoyment, foreign language boredom, perceived effectiveness

## Abstract

Self-regulated learning in technology-supported environments has attracted much scholarly attention in recent years. With the rapid expansion of online education, students’ emotions have also been studied extensively in second language acquisition. However, few empirical studies have examined the interrelationship between students’ self-regulated learning and emotions in the emerging field of language MOOCs (LMOOCs). This study bridged this gap by exploring the relationship between foreign language enjoyment (FLE), boredom (FLB), self-regulated learning (SRL), and perceived effectiveness in LMOOC learning. Data were collected among 356 successful learners of a language MOOC in mainland China through a cross-sectional study. The results showed that LMOOC learners had a high level of enjoyment and a moderate level of boredom. A significantly positive relationship was noted between FLE and SRL while a negative relationship was found between FLB and SRL. SRL was confirmed to be the mediator between FLE, FLB, and PE, which partially mediated the effects of FLE on PE and fully mediated the effects of FLB on PE. Perceived effectiveness was predicted by all SRL strategies and time management significantly predicted perceived effectiveness. The results provided pedagogical implications for students to develop positive emotions and effective SRL strategies to achieve better learning outcomes in LMOOC learning.

## 1. Introduction

MOOCs (Massive Open Online Courses) are interpreted as a heralding great change in institutional practices and the university system since their appearance over a decade ago (O’Connor, 2014), and have witnessed an unprecedented enrollment surge after the COVID-19 pandemic (Impey and Formanek, 2021). Language MOOCs, defined as “dedicated Web-based online courses for second languages with unrestricted access and potentially unlimited participation” (Bárcena and Martín-Monje, 2014), is an emergent and expanding field in foreign language education. Recent studies on LMOOCs from learners’ perspective mainly focused on their motivation, learning experience and academic performance (Henry and Marrs, 2015; Charbonneau-Gowdy, 2016; Sallam et al., 2020), whereas learner autonomy and strategy choices have received scant attention (Mısır et al., 2018; Ding and Shen, 2019). Self-regulated learning (SRL), which has typically been studied in face-to-face learning environments, has received increasing attention in the context of MOOCs. Self-regulated learning (SRL) is critical in MOOCs that “require high levels of learner autonomy and low levels of teacher presence” (Loizidou, 2021). The open and flexible nature of MOOCs places the onus on individual learners to create and navigate their own learning journey (Magen-Nagar and Cohen, 2017). Self-regulated learning (SRL) strategies, such as (meta)cognitive, motivational, and behavioral strategies use in the context of online language learning remain in need of further empirical inquiry (An et al., 2021). How learners adopt self-regulated learning in LMOOCs is still underestimated and our understanding of learners’ self-regulated learning strategies for target language learning is still limited. With the introduction of positive psychology, researchers are encouraged to expand the research scope of emotions to MOOCs learning for the reason that MOOCs learning presents challenges to students not only cognitively and socially, but also emotionally (Cleveland-Innes and Campbell, 2012). Emotions have been well studied in traditional language classroom settings (Dewaele, 2011; MacIntyre and Gregersen, 2012; Dewaele and MacIntyre, 2016), but little is known about how multiple emotions can function in online language learning contexts, such as LMOOCs.

Considering the effects of SRL strategies and emotions on the academic success of MOOCs learners (Davis and Nichols, 2016; Lee et al., 2020), recent studies have highlighted the necessity of understanding students’ SRL strategies and emotions when engaging with LMOOCs (Gafaro, 2019a,b). In previous empirical studies, self-regulated learning has been found to be positively related to learners’ academic performance and serves as a key derterminant of their success in MOOCs learning (Cohen and Magen-Nagar, 2016; Kizilcec et al., 2017). However, few empirical research to date have explored the relations between SRL, emotions and academic achievement in online language learning environments. Guided by the control-value theory of achievement emotions, this paper bridges this gap by exploring the influence of learners’ enjoyment, boredom and SRL strategies on their perceived effectiveness of LMOOCs. The interwoven relationships between these four factors are examined, as well as the mediating role of SRL strategies between students’ emotions and perceived effectiveness. The findings shed light on the insufficient research on L2 learners’ behavioral and psychological features in online EFL learning environments and offer insights for developing up-to-date technology-supported language teaching strategies. This paper sets out to contribute to the growing but still scarce body of research on informal and self-motivated language learning *via* massive open online courses (LMOOCs).

## 2. Literature review

### 2.1. Self-regulated learning

Self-regulated learning (SRL) has been generally acknowledged as the process in which learners employ (meta)cognitive, motivational, behavioral and emotive strategies to control and regulate their learning (Zimmerman, 1990; Perry and Rahim, 2011; Panadero, 2017; Wandler and Imbriale, 2017). Self-regulated learning has made a major contribution to educational psychology in the past decades. SRL studies have developed and expanded into a comprehensive theoretical framework covering several theoretical models and methods from different perspectives (Sitzmann and Ely, 2011; Zimmerman and Schunk, 2011). Panadero (2017) analyzed and compared six main SRL models and found that these models form an integrative and coherent framework. Although different models present significant differences in the conceptualization of SRL, there is a consensus that SRL is a cyclical process which is composed of different phrases (e.g., preparatory, performance, and appraisal phrases) and they all explore SRL from three main areas, including (meta)cognition, motivation and emotion (Panadero, 2017).

### 2.2. Self-regulated learning in MOOCs

Compared with face-to-face learning and synchronous online learning, MOOCs learning is more open and self-paced, which shifts the control of learning from educators to individuals (Fournier et al., 2014; Wong et al., 2019). High dropout rates in MOOCs learning have been a challenging issue and the lack of guidance from teachers accounts for this phenomenon as one of the main reasons (Hew and Cheung, 2014). MOOCs learners are expected to have a greater ability to regulate their own learning because there is a lack of support or guidance from instructors (Hood et al., 2015). Online self-regulated learning strategies are proven to be predictors of students’ perceived usefulness of online learning activities (Shih et al., 2019), and students’ better online behavioral and cognitive engagement benefited from their use of SRL strategies (Guo et al., 2021). Previous studies into the role of self-regulated learning in MOOCs have found learners’ self-regulated learning strategies are positively related to their engagement and academic performance (Kizilcec et al., 2017; Magen-Nagar and Cohen, 2017). Albelbisi et al. (2021) revealed that self-regulated learning strategies are a key determinant of MOOC success based on a structural equation modeling analysis. Yılmaz and Yılmaz (2022) investigated learners’ self-regulated strategies in MOOCs learning and found that students using the smart MOOC environment had high self-regulation skills. To sum up, self-regulated learning has been regarded as the key to successful MOOCs learning.

### 2.3. Self-regulated learning in language MOOCs

LMOOCs have experienced an exponential growth since their appearance in 2012 (Jitpaisarnwattana et al., 2019; Akram et al., 2021). Since the outbreak of the COVID-19 pandemic, language learners in many countries have resorted to LMOOCs as a new channel to continue their language learning and has led language learning to be in the top 10 subjects of interest in MOOCs (AlQaidoom and Shah, 2020; Martín-Monje and Borthwick, 2021). Language MOOCs differ from other MOOCs in that instructional videos are not only a way of lecturing, but also a source of authentic language input and an opportunity for students to engage themselves in the target language and culture (Sokolik, 2014). Therefore, LMOOCs learners need to employ flexible strategies to choose the most appropriate language learning content, language teaching approach and method, which gives more room for their exercise of self-regulated learning. However, little attention has been paid to self-regulated learning in LMOOCs. Early attempts to look into learners’ planning, control and management of LMOOCs learning focus on the explorations of learner autonomy. Read and Rodrigo (2014) stated that language MOOCs might be “challenging for students who are not used to studying in such an autonomous manner” (p. 93). Rubio (2014) compared students’ academic success in a face-to-face language course and an LMOOC, finding that the large effect size in LMOOCs contributed to the improvement of their learner autonomy. Ding and Shen (2020) interviewed 38 learners of an English vocabulary MOOC, highlighting the complexity of learner autonomy in which learners adopted a variety of metacognitive, motivation control and emotion control strategies. In recent three years, scholars started to expand their attention from learner autonomy to SRL in LMOOCs. Gafaro (2019a,b) found the positive influence of LMOOCs on the improvement of students’ SRL strategies and emphasized the necessity of understanding students’ SRL strategies when integrating LMOOCs into formal language courses. Some recent studies also demonstrated the supportiveness of LMOOCs for enhancing students’ SRL either in commercial language MOOCs (Zhang et al., 2021) or in blended language learning (Loizidou, 2021). L2 learning is a multifaceted phenomenon whose process and performance are both affected by learner-internal and learner-external factors. Although students’ self-regulated learning and autonomy have been explored in LMOOCs, their connections with students’ sociopsychological factors, such as emotions, remain largely unanswered. This paper aims to address this gap by exploring the links between students’ enjoyment, boredom, SRL and perceived effectiveness in LMOOC learning.

### 2.4. Foreign language enjoyment

Pekrun et al. (2007) defined enjoyment as “a sense of exhilaration arising in the face of a new, complicated, and challenging activity that arouses interest.” Dewaele and Mac Intyre (2014) first introduced enjoyment as a positive counterpart to second language acquisition, namely foreign language enjoyment (FLE). FLE research flourish with the growing popularity of positive psychology in the field of SLA and has become one of the most studied positive emotions (Jiang and Dewaele, 2019). As for the inner structure of FLE, Dewaele and Mac Intyre (2016) explored the constructs underlying 21 FLES items with a principal component analysis, which yielded a two-factor solution about FLE, FL enjoyment-social, and FL enjoyment-private. They later complemented the results, indicating that the structure of FLE is culture-based (Li et al., 2018). Jin and Zhang (2021) found that there were three dimensions underneath the construct of FLE, namely Enjoyment of Teacher Support, Enjoyment of Student Support, and Enjoyment of Foreign Language Learning.

Enjoyment has become one of the most studied positive emotions (Jiang and Dewaele, 2019) as a focal point within the positive emotional network (Piniel and Albert, 2018). There are abundant studies focusing on the relationship between FLE and learner-internal factors, such as emotions, motivation, mindset and learning engagement. Dewaele and Mac Intyre (2014) were among the first to conduct a series of empirical studies on FLE in coexistence with other emotions. In recent years, FLE has been mostly studied in association with anxiety (Dewaele and Alfawzanet, 2018; Dewaele and Dewaele, 2020; Pan and Zhang, 2021) and boredom in foreign language classroom teaching (Li and Dewaele, 2020; Kruk et al., 2022). Besides emotions, it is also found that students’ enjoyment in foreign language learning is positively related to learners’ motivation, self-efficacy, and growth mindset (Zhang et al., 2020; Karlen et al., 2021; Hu et al., 2022; Zhang and Dong, 2022). FLE was also recognized as a facilitator of students’ learning engagement, a catalyst for interest in the online learning environment (Zhao and Yang, 2022), and a mediator between trait emotional intelligence and learning achievement (Dewaele and Alfawzan, 2018; Li, 2020; Wang and Xu, 2021). Recent studies also proved that enjoyment was related to learner-external factors, such as teaching content, classroom environment, teacher enthusiasm, friendliness and teacher support (Dewaele and Li, 2021; Shao and Parkinson, 2021; Zhao and Yang, 2022). In this study, we explored the influence of the online classroom environment on students’ enjoyment in LMOOC learning and its relationship with boredom, self-regulated learning and self-perceived effectiveness.

### 2.5. Foreign language boredom

Boredom is an unpleasant academic emotion associated with certain distorted time perceptions and certain tendencies such as “disengagement,” “inattention,” and “time dragging” (Putwain et al., 2018; Li et al., 2020). Based on boredom research in general educational contexts, Li et al. (2021) proposed the construct of foreign language learning boredom (FLLB) and conceptualized it as a three-dimensional achievement emotion. In their subsequent studies, “FLLB” was used interchangeably with “FLB” (Foreign Language Boredom) and “FLCB” (Foreign Language Classroom Boredom) in studies of Li et al. (2022) and Li and Han (2022). In this study, to keep consistent with the term “FLE,” we used “FLB” to represent foreign language boredom in online language learning contexts.

Regarded as a “silent emotion” (Pekrun et al., 2010), FLB remains to be an area relatively under-researched but has been found to permeate L2 classes in different learning contexts. Chapman’s (2013) study of boredom in German classes is the start of the exploration of foreign language boredom in the field of Applied Linguistics. Most of the attention is drawn to mainly two fields: inner structure and dynamic changes of FLB, and the relationship between FLB and other psychological well as environmental factors, such as willingness to communicate, and motivation (Pekrun et al., 2007; Fahlman et al., 2009; Li et al., 2021; Kruk et al., 2022). Li and Dewaele (2020) focused on FLB in online English learning and develop a 7-factor foreign language learning boredom scale. Additionally, the antecedents and consequences of boredom are explored externally and internally. Zawodniak et al. (2017) investigated the relationship of boredom with activities, content subjects, language classes, and teacher behaviors. They pointed out that inadequate devotion of teachers, monotonous realia, boring learning content, and lack of meaning for learning are the main reason for students’ boredom in language class Pawlak et al. (2020). Pawlak et al. (2020) consolidated and supplemented the theory by claiming that the more relevant the tasks are to students’ real lives, the lower level of boredom they would have. From the view of psychology, attentional theory of boredom proneness (LePera, 2011) holds a view that the boredom experienced is a result of students’ unmet expectations and interests. Moreover, Kruk (2022) introduces the concept—willingness to communicate—as an antecedent of foreign language boredom. As for the consequences of FLB, Wang and Xu (2021) investigated trait boredom and state boredom, concluding that boredom harms foreign language learning, which weakens individual participation and leads to a lack of attention. The present study takes a holistic view to investigate the effects of enjoyment and boredom on EFL learners’ self-regulated learning and self-perceived effectiveness in LMOOC learning.

### 2.6. The relationship between FLE, FLB, SRL, and perceived effectiveness

The control-value theory of achievement emotions proposed by Pekrun (2006) offers an integrative framework for analyzing the antecedents and effects of emotions experienced in achievement and academic contexts. With a growing number of studies separately exploring the antecedents and outcomes of students’ achievement emotions, researchers have borrowed the control-value theory (CVT) from general educational psychology to foreign language learning to provide a comprehensive understanding of L2 students’ achievement emotions (e.g., Shao et al., 2020; Li, 2021; Li and Wei, 2022). CVT suggests that achievement emotions are closely linked to appraisals of achievement-related control and value. When students feel in control over their learning and value achievement, positive emotions such as enjoyment, hope, and pride are promoted, and negative emotions such as anxiety, boredom, and hopelessness are reduced. The theory also holds that academic emotions involved with the students’ use of learning strategies and self-regulation eventually influence their achievement. In this study, CVT is applied as a theoretical model to examine the mediating effects of self-regulated learning on the relationship between FLE, FLB and perceived effectiveness in LMOOC learning.

A series of studies have demonstrated the role of emotion in SRL assuming that positive emotions enhance students’ self-regulated learning and negative emotions facilitate reliance on external guidance (Pekrun et al., 2007, 2010). Their findings have shown that enjoyment, hope, and pride are positively related to self-regulated learning, whereas hopelessness and boredom relate negatively to self-regulated learning (Linnenbrink, 2007; Pekrun et al., 2011). Self-regulated learners are reported to have positive emotions, including hope, enjoyment, and pride in learning and they control and regulate negative emotions, such as anger, anxiety, boredom, and frustration (Pekrun, 2006; Pekrun et al., 2010). Based on a survey of 5,805 undergraduate students, Mega et al. (2014) developed a structural equation model showing that students’ emotions influence their self-regulated learning and their motivation, and in turn, affect academic achievement. Karlen et al. (2021) also reported that students’ self-concepts about SRL are positively related to their enjoyment and academic achievements.

Perceived effectiveness refers to a general “evaluation of the overall effectiveness of the course” (Peltier et al., 2003). It has played a vital role in enhancing MOOC effectiveness by predicting learner retention for MOOCs (Sujatha and Kavitha, 2018) and has served as a measure of learners’ satisfaction with online learning environments (Bolliger and Halupa, 2012; Hone and El Said, 2016). Since it is not practical to measure hundreds of thousands of students’ learning behaviors in MOOCs (Jung et al., 2019), researchers have paid more attention to perceived effectiveness as a means to determine learning outcomes in MOOCs learning (Gamage et al., 2015; Lee et al., 2020). Previous research findings showed that SRL strategies predicted perceived effectiveness in online learning environments (Lee et al., 2020). Considering that successful MOOC learners were more capable of self-regulating their learning and showed significantly higher levels of perceived effectiveness (Davis and Nichols, 2016), this study explores successful MOOC learners’ FLE, FLB, and self-regulated learning as well as their relationship with perceived effectiveness in LMOOC learning.

With the wide spread of web-based communication technologies, more scholarly attention has been paid to students’ emotional states in online language learning environments (Loderer et al., 2020). Li and Dewaele (2020) pointed out that EFL students experience more boredom due to the lack of real interactions with teachers or other peer learners. Li and Han (2022) explored the effects of foreign language enjoyment, boredom and anxiety on their online English classroom learning. Beirne et al. (2018) found that positive emotions, such as curiosity, excitement and pride, were reported most strongly by participants throughout LMOOC learning. However, how these emotions are combined together to exert effects on their self-regulated learning and in turn influence their academic achievement in online language learning have not been investigated. Meanwhile, although self-regulated learning has been widely investigated in traditional classroom settings, few empirical studies have been conducted in MOOCs learning and little is known about how SRL strategies is used by L2 learners in learning LMOOCs. The lack of research on the relationships between emotions, SRL strategies and perceived effectiveness in LMOOCs learning and the need to be better prepared for asynchronous online language teaching in post-pandemic era are the motivations for this study. The present study, to our best knowledge, is the first to investigate the relationship between academic emotions, SRL strategies and perceived effectiveness in LMOOC learning. This study contributes to the existing literature on LMOOCs by providing an in-depth understanding of the cognitive and metacognitive mechanisms underlying online L2 learning. The findings also shed insights into the design and implementation of successful LMOOCs by integrating effective emotional intervention and SRL strategies.

Drawing on the control-value theory and previous studies on emotions in online language learning as well as SRL strategies in MOOCs learning, we form our hypotheses that FLE is positively correlated with learners’ SRL strategies and perceived effectiveness; while FLB is negatively correlated with SRL strategies and perceived effectiveness. This study also hypothesized that SRL strategies has mediating effect between learners’ feelings of emotions and learning outcomes. Our research aims to address the following research questions:

1. What are the relationships between foreign language enjoyment, foreign language boredom, self-regulated learning, and perceived effectiveness in LMOOC learning?
2. How do different SRL strategies contribute to students’ perceived effectiveness in LMOOC learning?
3. How do SRL strategies mediate between foreign language enjoyment, boredom and students’ perceived effectiveness in LMOOC learning?

## 3. Methodology

### 3.1. Participants

Our participants enrolled in an English MOOC titled “An Impression of British and American Culture” which was delivered in English. This LMOOC aims to improve students’ general knowledge of British and American culture, developing their intercultural communication competence as well as basic English skills. The online course consists of 12 chapters and all the learning materials, including lecture videos, readings, and quizzes, can be obtained from China’s biggest MOOC platform “icourse163.org.” It generally takes students 12–14 weeks to finish learning this LMOOC. This English MOOC was provided to the students as a complementary teaching resource for the course “An Introduction to English-speaking countries,” which is a required course for English majors. Students are suggested to finish learning one chapter each week, but there is no strict time limit. Therefore, students feel free to complete all the assignments according to their preferred pace within the semester. Students’ performance in learning the LMOOC is considered as a reference for judging students’ academic performance in learning the local required course. This study adopted a cross-sectional survey and students needed to finish a questionnaire survey about their emotions, self-regulated learning and perceived effectiveness in learning the LMOOC at the end of the semester. Before the data collection, students were informed of the purpose of the study and could choose to participate or not according to their own will. They were assured that their responses would remain strictly confidential and only be used for research purposes. A total of 356 Chinese undergraduate students participated in the present study. The participants included 198 sophomores and 158 junior students majoring in English from two universities in the south of China. The number of valid questionnaires was 301 and the data from the students who did not complete the LMOOC learning or those who failed to finish the questionnaire were excluded. These participants consisted of 107 male students (30.1%) and 282 female students (69.9%). The students’ age ranged from 18 to 20, with a mean age of 19.33 (SD = 0.501). The demographic information of the participants is shown in Table 1.

**Table 1:** Participants’ demographic information (*N* = 301).

School	Male	Female	Sophomore	Junior Student	Mean age
A	48	103	87	72	19.21
B	49	101	81	61	19.45
Total	97	204	168	133	19.33

### 3.2. Instruments

The questionnaire consisted of two parts. The first part asked for students’ demographic information, including their age, gender and grade in university. The second part consisted of four scales aiming at measuring students’ enjoyment, boredom, self-regulated learning strategies and perceived effectiveness in their LMOOC learning. The scales were all derived or adapted from the existing literature to ensure content validity. Considering that Chinese university students’ English proficiency may not be sufficient to understand the items originally in English, four bilingual (Chinese and English) research assistants translated the questionnaires to make sure Chinese students can understand the meaning of each item. Detailed information on the four scales is presented as follows.

#### 3.2.1. Foreign language enjoyment scale

Students’ enjoyment in LMOOC learning was measured through the 11-item Chinese version of Foreign Language Enjoyment Scale adapted by Li et al. (2018). Some items were deleted and reworded to better suit the conditions of LMOOCs learning in China. The final four items were scored on a 5-point Likert scale, ranging from ‘1 (disagree completely)’ to ‘5 (agree completely)’. The Confirmatory Factor Analysis (CFA) was performed to confirm the one-factor structure of the questionnaire and good model fits were found with *χ*^2^/df = 4.85 (*p* < 0.001), CFI = 0.97, TLI = 0.94, SRMR = 0.03. The Cronbach’s α for the single-factor questionnaire was 0.87, which was considered excellent for adequate measurement.

#### 3.2.2. Foreign language classroom boredom subscale

Students’ boredom in LMOOC learning was measured by the revised version of Foreign Language Classroom Boredom Subscale (FLCBS) designed by Li et al. (2020). The subscale questions measured the participants’ proneness or disposition toward boredom and recurrent experience of boredom in learning English online, which originally came from the Foreign Language Boredom Scale (FLBS) (Li et al., 2020). The subscale was adapted according to the online learning environment, which had four items and was scored by a 5-points Likert scale, ranging from ‘1 (disagree completely)’ to ‘5 (agree completely)’. The FLCBS was tested to be a psychometrically sound tool, exhibiting excellent validity and reliability for each of its subscales (Li, 2020). It was reported that FLCBS had a desirable construct validity (χ^2^/df = 3.79; *p* < 0.001; CFI = 0.99; TLI = 0.98; SRMR = 0.011; RMSEA = 0.08) as well as a good reliability (Cronbach’s alpha = 0.91).

#### 3.2.3. Online self-regulated learning questionnaire

This study adopted The Online Self-Regulated Learning Questionnaire (OSLQ) to assess students’ SRL strategies in LMOOC learning. Formulated by Barnard et al. (2009) and translated into a Chinese version by Fung et al. (2018), the questionnaire consisted of 24 statements and used a 5-point Likert scale ranging from ‘1 (disagree completely)’ to ‘5 (agree completely)’. Items were adapted and reworded to better suit for LMOOCs learning context. Two professional academics specialized in MOOCs learning and SRL were invited to assess the face and content validity of each item. Fourteen items were finally retained to investigate Chinese students’ SRL strategies in learning language courses online. Exploratory factor analysis (EFA) was used to analyze the data of the pre-test. The Bartlett’s spherical test provided a chi-square value of 2151.29 (*p* < 0.001), and the Kaiser-Meyer-Olkin (KMO) statistic was 0.94, indicating that the data were suitable for structure detection (Kaiser, 1958). Finally, a three-factor structure with 11 items was extracted, which accounts for 74.13% of total variance. The three factors were learning strategies (6 items), time management (3 items), and goal setting (2 items). Learning strategy (LS) refers to students-initiated behaviors while learning, including taking notes, marking out questions, and seeking help from teachers or classmates. Time management (TM) refers to the students-initiated management of the time length, time period, and time interval to learning LMOOCs. Goal setting (GS) refers to student-initiated setting of educational goals or subgoals and planning for sequencing, timing, and completing activities related to these goals. The data obtained from 301 participants showed that the 11-item three-factor structure of OSLQ had a desirable construct validity (χ^2^ /df = 3.29, p < 0.001, CFI = 0.96, TLI = 0.94, SRMR = 0.041, RMSEA = 0.09) and reliability (Cronbach’s alpha = 0.89). Standardized factor loadings for CFA were all above 0.7 and the Cronbach’s alpha coefficients for three factors were: 0.91 for learning strategies, 0.77 for time management, and 0.7 for goal setting.

#### 3.2.4. Online learning perceived effectiveness scale

Students’ perceived effectiveness of learning LMOOCs was measured through the online learning perceived effectiveness scale adapted from the online learning effectiveness scale designed by Shen and Wu (2020). The one-factor scale included six items using a scale from “1 (disagree completely)” to “5 (agree completely)”. The scale was proved to be a sound tool, exhibiting high reliability (Cronbach’s alpha = 0.86) and acceptable validity (*χ*^2^ /df = 3.75, *p* < 0.001, CFI = 0.98, TLI = 0.96, SRMR = 0.025, RMSEA = 0.09).

### 3.3. Data analysis

Survey datasets of this study were analyzed using SPSS 26 and AMOS 26. Firstly, descriptive analysis and normality tests were conducted for each observed variable. To answer the first research question, three linear regression analyses and one multiple regression analysis were run with the three types of SRL strategies as predictors and perceived effectiveness as the outcome variable. Pearson product–moment correlation was carried out to assess the relationship between FLE, FLB, SRL strategies, and perceived effectiveness. The data were analyzed using the Statistical Product and Service Solution 26.0 (SPSS 26.0) and AMOS 26.0 to investigate the correlations between variables and structural equation modelling revealed the complex relationships between variables in this study. The model fit indices were interpreted using several model fit indicators (Schermelleh-Engel et al., 2003): *χ*^2^/df ratio value (should be lower than 3), the root mean square error of approximation (RMSEA, should be ranged between 0.05–0.08), the comparative fit index (CFI, should be higher than 0.9), Tucker-Lewis Index (TLI) (a value equal to or greater than 0.90 indicates acceptable model fit), and the standardized root mean square (SRMR, should be lower than 0.08). Additionally, a maximum-likelihood (ML) estimation was used for estimation, and a bootstrapping method with a 95% confidence interval was used to test indirect effects in this study (Mac Kinnon et al., 2004).

## 4. Results

Before answering our research questions, descriptive statistics of students’ foreign language enjoyment, boredom, SRL strategies, perceived effectiveness are presented in Table 2. Students reported a high level of FLE (mean of FLE = 4.01) and a moderate level of FLB in LMOOC learning (mean of FLB = 2.47). Both students’ SRL and strategies PE show a moderate level (mean of SRL = 3.48, mean of PE = 3.59). Among the three SRL strategies, time management (TM) was used most frequently than learning strategy (LS) and goal setting (GS) (*M* = 3.67, SD = 0.83). In Table 2, the correlations between FLE, FLB, SRL, LS, TM, GS, and PE were also listed. A normality test of the data was carried out as well. Absolute values of skewness and kurtosis were all smaller than 2 and 7, respectively, suggesting no violation of normality assumptions.

**Table 2:** Descriptive statistics and correlation coefficients between the latent variables (*N* = 301).

Variables	Mean	SD	1	2	3	4	5	6	7
FLE	4.01	0.79	1.00						
FLB	2.47	1.04	−0.65^**^	1.00					
SRL	3.38	0.81	0.61^**^	−0.52^**^	1.00				
LS	3.22	0.91	0.55^**^	−0.46^**^	0.96^**^	1.00			
TM	3.67	0.83	0.58^**^	−0.54^**^	0.87^**^	0.73^**^	1.00		
GS	3.41	0.92	0.56^**^	−0.46^**^	0.84^**^	0.71^**^	0.72^**^	1.00	
PE	3.59	0.79	0.59^**^	−0.47^**^	0.63^**^	0.57^**^	0.62^**^	0.55^**^	1.00

M, mean, SD, standard deviation.

**p < 0.01. FLE, foreign language enjoyment; FLB, foreign language boredom; SRL, self-regulated learning strategies; LS, learning strategies; TM, time management; GS, goal setting; PE, perceived effectiveness.

To answer the first research question, Pearson correlation analysis reveals the correlations between variables in this study. For the estimation method, a maximum likelihood estimation was selected. The results showed that the four variables under investigation significantly correlated with each other (see Table 3). As expected, FLE positively correlated with SRL strategies and PE (rs = 0.55–0.61, ps < 0.01) but demonstrated a negative correlation with FLE. However, FLB negatively correlated with FLE, SRL strategies and PE (rs = 0.46–0.54, ps < 0.01). Positive correlations were found between SRL strategies and perceived effectiveness (r = 0.63, *p* < 0.01). All SRL strategies (LS, TM, and GS) were significantly positively correlated with perceived effectiveness (rs = 0.55–0.62, ps < 0.01), among which time management had the strongest correlations with PE.

**Table 3:** Regression models of SRL strategies as predictors for perceived effectiveness (*N* = 301).

Regression equations		Fit index			Coefficient	
Predictor	Outcome	*R* ^2^	Adjusted *R*^2^	*F*	*β*	SE (*B*)
LS	Perceived effectiveness	0.33	0.32	144.49	0.57^****^	0.03 (0.41)
TM		0.38	0.38	183.03	0.62^****^	0.07 (0.98)
GS		0.31	0.31	132.45	0.55^****^	0.10 (1.19)
LS TM GS		0.42	0.42	72.12	0.15^**^	0.05 (0.15)
					0.36^****^	0.11 (0.57)
					0.20^***^	0.15 (0.33)

**p < 0.05; ***p < 0.01; ****p < 0.001.

Concerning the second research question, a multiple regression analysis (stepwise method) was conducted to further investigate the contributions of SRL strategies to students’ perceived effectiveness in LMOOC learning. All the variance inflation factors were between 2 and 5. According to Gujarati (1995), if the value of VIF is 10 and above, the variables are said to be collinear. Therefore, no clear evidence of multicollinearity was detected. As shown in Table 3, LS (β = 0.57, *p* < 0.001), TM (β = 0.62, p < 0.001), GS (β = 0.55, p < 0.001) had a statistically significant influence on perceived effectiveness. When the three SRL strategies entered the equation simultaneously, it turned out that TM (β = 0.36, *p* < 0.001) was the most significant predictor of the perceived effectiveness of LMOOC learners. The influences of GS (β = 0.20, p < 0.01) and LS (β = 0.15, *p* < 0.05) on perceived effectiveness were also significant though weaker than that of TM.

**Figure 1:**
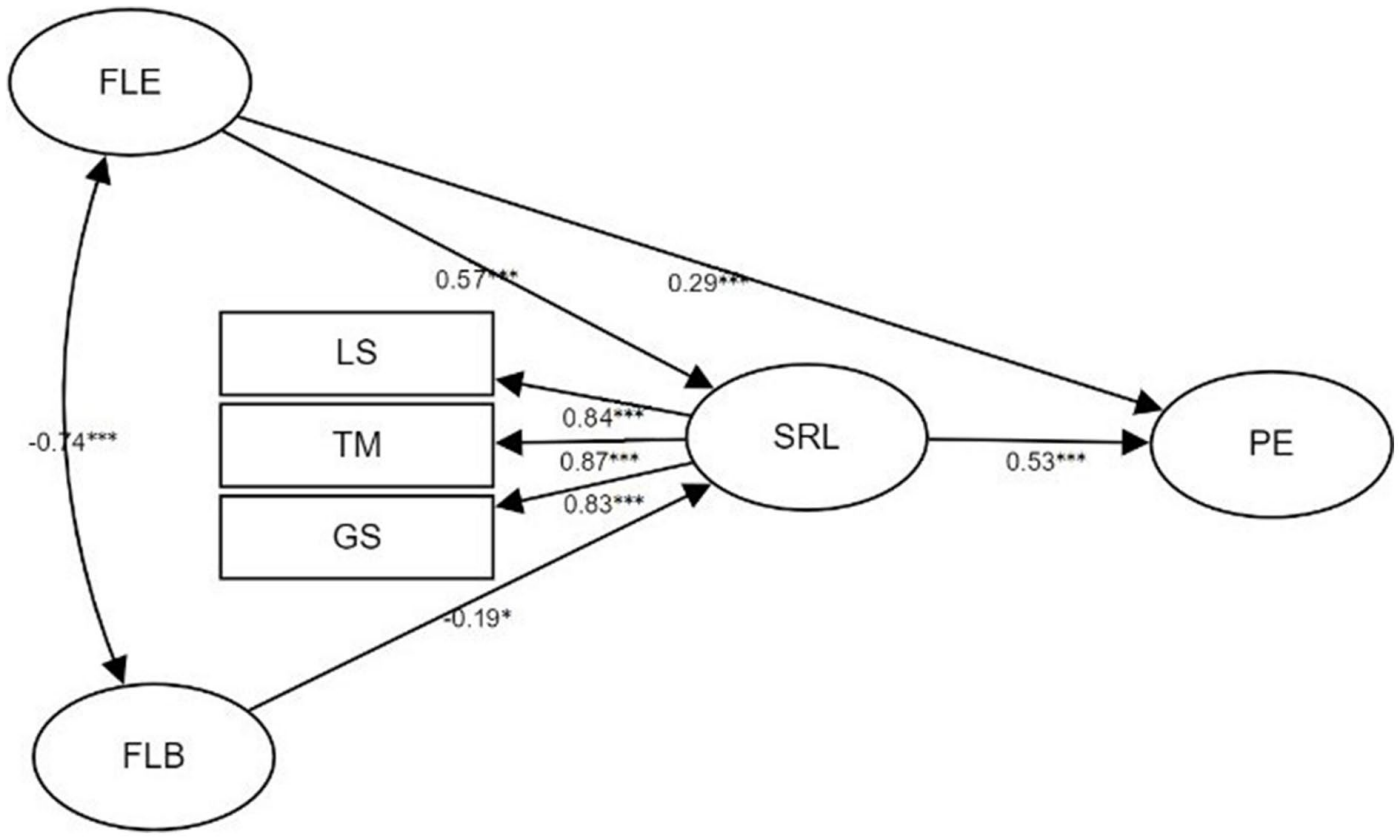
Structural equation modal of the relationship between FLE, FLB, SRL, and PE. *p < 0.05, **p < 0.01, ***p < 0.001. FLE, foreign language enjoyment; FLB, foreign language boredom; SRL, self-regulated learning strategies; LS, learning strategies; TM, time management; GS, goal setting; PE, perceived effectiveness.

To further investigate the relationship between the four variables, a structural equation model (SEM) was performed and the standardized path coefficients were presented in Figure 1. A revised model was proposed, which deleted the insignificant path and showed an excellent goodness of fit (*χ*^2^/df = 2.640, *p* < 0.001, TLI = 0.944, CFI = 0.954, RMSEA = 0.074, SRMR = 0.045). In accordance with the correlation analyses (Table 1), FLE was statistically significantly associated with both SRL (β = 0.57, p < 0.001) and PE (β = 0.26, p < 0.001). Also, the association between SRL and PE was found statistically significant (β = 0.53, p < 0.001). FLB had no significant direct association with PE and it was negatively linked with SRL strategies, though not as significant as other paths. These variables explained 58% of the variances in perceived effectiveness.

To answer the third research question, we tested the mediating effects of SRL using a bootstrapping method proposed by Preacher and Hayes (2008). The result and a bootstrapped 95% confidence interval were reported in Table 4. The indirect effects of FLE and FLB on PE, respectively, *via* SRL were equal to the product of the coefficients of each path in their mediation chains (i.e., FLE: 0.57*0.53 = 0.3; FLB: −0.19*0.53). If 0 was not included in the 95% confidence interval, the path was confirmed significant (Chen et al., 2013). The 95% confidence intervals did not include 0 neither, manifesting that the effects were significant. Considering that FLE had significant direct effects on PE, SRL only partially mediated the influence of FLE on PE (i.e., 0.3/0.59 = 58.85%). On the other hand, since the direct effects of FLB on PE were not significant, SRL played a fully mediating role in the relation between FLB and PE. In comparison, FLE had greater total effects than FLB. These variables explained 53% of the variance in LMOOC learners’ PEs.

**Table 4:** Standardized direct, indirect, and total effects for structural model.

Type	Estimates	SE	*Z*	Bias-corrected 95% CI
Total effect of FLE	0.59	0.061	9.67	0.362 ~ 0.602
Total direct effect of FLE	0.29	0.062	4.68	0.162 ~ 0.403
Total indirect effect of FLE	0.30	0.056	5.36	0.095 ~ 0.311
Total (indirect) effect of FLB	−0.10	0.035	−2.85	−0.166 ~ −0.028

**Figure 2:**
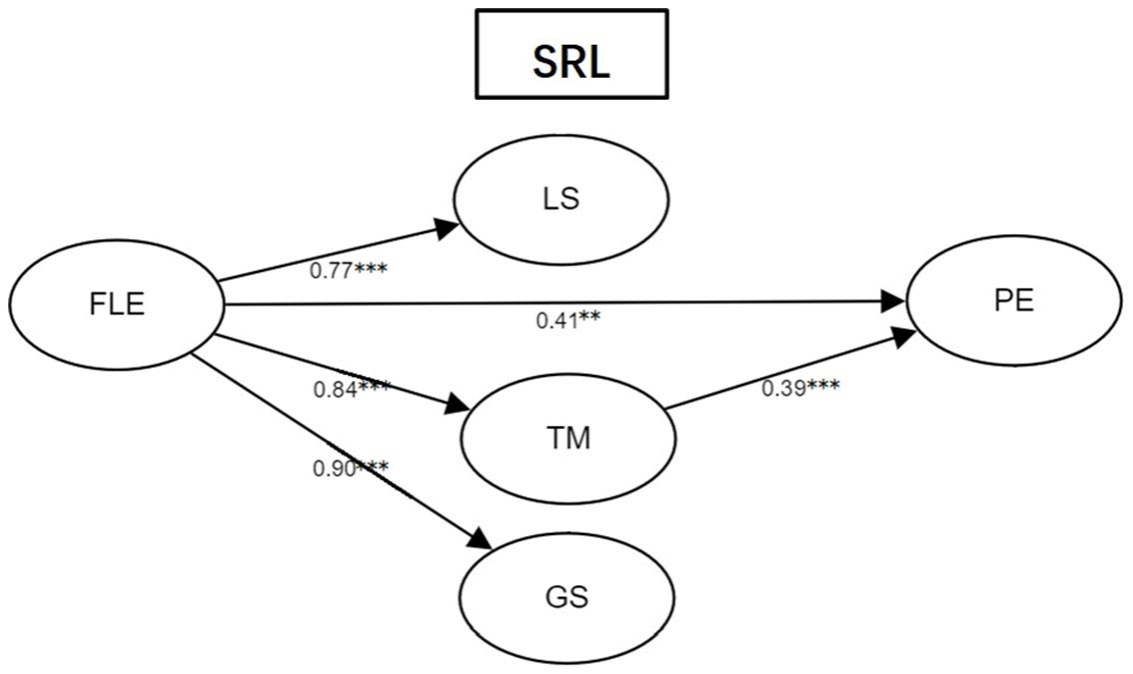
Structural equation modal of the relationship between FLE, LS, TM, GS, and PE. **p < 0.01, ***p < 0.001. FLE, foreign language enjoyment; LS, learning strategies; TM, time management; GS, goal setting; PE, perceived effectiveness.

**Figure 3:**
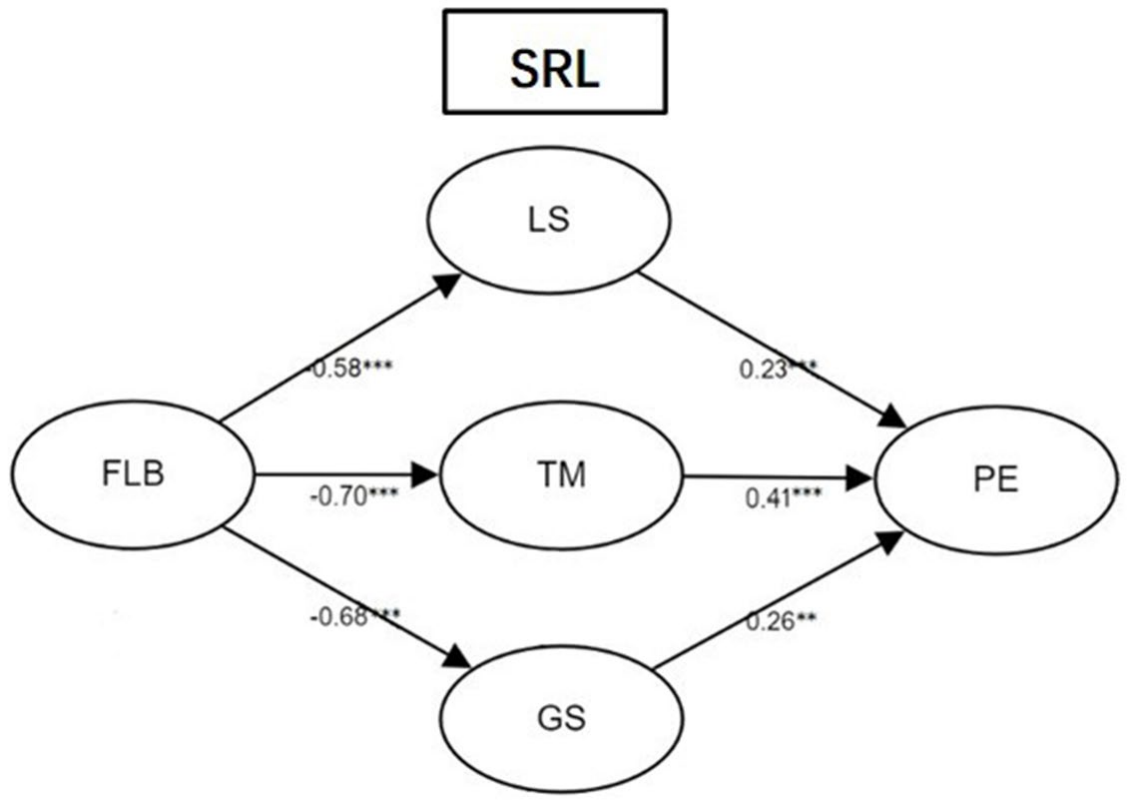
Structural equation modal of the relationship between FLB, LS, TM, GS, and PE. **p < 0.01, ***p < 0.001. FLB, foreign language boredom; LS, learning strategies; TM, time management; GS, goal setting; PE, perceived effectiveness.

Considering that FLE and FLB were latent factors, we continued to use SEM to figure out the mediating effects of different SRL strategies on FLE and FLB. The mediating effects of FLE and FLB were investigated in two separate models and the results were presented in Figures 2, 3. In Figure 2, the modal showed acceptable goodness of fit (*χ*^2^/df = 2.25 < 3, TLI = 0.84, CFI = 0.88, RMSEA = 0.10). FLE was statistically significantly associated with LS (β = 0.77, *p* < 0.001), TM (β = 0.84, *p* < 0.001), and GS (β = 0.90, *p* < 0.001). The mediating effects of LS and GS were not statistically significant and only TM served as the mediator between FLE and PE. One of the possible explanations for the results is that the pleasing experience in LMOOC learning could attract learners to spend more and more time in learning activities. The indirect effect of FLE on PE *via* TM was 0.84*0.39 = 0.33 (*p* < 0.001). The 95% bias-corrected confidence interval for the mediating effect was between 0.054 and 0.27 and the standard error was at 0.13, indicating that the mediating effects in this chain were significant. FLE was also directly linked with PE (β = 0.41, *p* < 0.01). Hence, the indirect effects of FLE were partially mediated by TM (i.e., 0.33/(0.41+ 0.33) = 44.59%). The model explained 58% of the variances in PE.

Figure 3 demonstrated that three SRL strategies serve as mediators between FLB and perceived effectiveness in LMOOC learning. The significant indirect effects of LS and GS were − 0.58*0.23 = −0.13 (*p* < 0.001) and − 0.68*0.26 = −0.17 (*p* < 0.01). FLB had the greatest indirect effects through TM, which was −0.28 (*p* < 0.001). 95% bias-corrected confidence interval of three mediators did not include 0, further indicating that the indirect effects were statistically significant. Since there was no direct effect between FLB and PE, three SRL strategies were full mediation variables between FLB and PE. The model explained 51% of the variances in perceived effectiveness of LMOOC learning.

## 5. Discussion

The present study investigated the relationship between foreign language enjoyment, boredom, self-regulated learning strategies and perceived effectiveness. Although abundant studies have explored academic emotions in foreign language classrooms, little empirical research has unveiled the relations between emotions, self-regulated learning and academic achievement in online language learning environments. The three objectives of this study are, first, to explore the interwoven relationship between these four factors; second, to examine the effects of different SRL strategies on student’s perceived effectiveness; and finally, to determine whether SRL strategies mediate the relationship between foreign language enjoyment, boredom and perceived effectiveness.

The descriptive analysis showed a general condition of EFL students’ enjoyment, boredom, self-regulated learning and perceived effectiveness in LMOOC learning. As two academic emotions that are commonly experienced in traditional L2 classes, FLE and FLB in the online learning context, especially in LMOOCs featured by insufficient human interaction and feedback to the learner’s written and oral production (Loizidou, 2021), remain under-researched. In this study, a relatively high level of FLE (M = 4.01, SD = 0.79) and a moderate level of FLB (M = 2.47, SD = 1.04) were reported by 301 LMOOC learners in China, confirming assumptions about the pervasiveness of the two emotions. The finding echoed prior research results of Dewaele and Mac Intyre (2014) and Li et al. (2021) that positive and negative emotions can exist together in language learning contexts. Similar findings appeared in Beirne et al.’s (2018) study which found that LMOOCs learners experienced both positive and negative emotions but positive emotions were reported most strongly by learners. Li and Han (2022) also concluded that Chinese EFL students experienced a high level of enjoyment (*M* = 3.59, SD = 0.60) and a moderate level of boredom (*M* = 2.62, SD = 0.86) in online English classrooms during the COVID-19 pandemic. Similarly, Wang and Li (2022) study reported that learners had a relatively high level of FLE (*M* = 3.91) and a moderate level of FLB (*M* = 2.33) when they transformed a face-to-face English course to online teaching. Our study suggested that students could perceive a higher level of enjoyment and a lower level of boredom in learning LMOOCs than in the online language classrooms of conventional courses. Students’ enjoyment in learning LMOOC is related to the special features of MOOCs being “adaptations of conventional courses adopting the same procedural metaphors as face-to-face courses but allowing independent and flexible learning” (Hood et al., 2015).

Meanwhile, students reported a moderate level of both self-regulated learning strategies (*M* = 3.38, SD = 0.81) and perceived effectiveness (*M* = 3.59, SD = 0.79) in LMOOC learning. Previous studies showed divergent findings on students’ use of self-regulated strategies in various online language learning contexts. An et al. (2021) reported a high level of technology-assisted SRL strategies (*M* = 4.25, SD = 1.13) of students in online English learning and technology-based vocabulary learning was reported to be highly frequently used among all strategies. In Lin et al.’s (2021) study, international students reported a moderate level of SRL strategies (*M* = 3.49, SD = 1.36) in learning Chinese MOOCs. It indicates that students’ SRL strategies in online language learning vary in different contexts and a moderate level of SRL strategies was found in previous language MOOCs studies.

In an earlier study of Hui et al. (2008), students showed a moderate level of perceived effectiveness in technology-assisted language learning (*M* = 3.24, SD = 1.11). Lee et al. (2020) also reported a moderate level of students’ perceived effectiveness in MOOCs learning (*M* = 3.45, SD = 0.38). The limited previous research on MOOCs learners’ perceived effectiveness demonstrated that they experienced not very high but acceptable satisfaction with MOOCs learning. The reasons for their moderate level of SRL strategies and perceived effectiveness need to be further explored in both quantitative and qualitative studies taking more contextual factors into consideration.

Concerning the first research question, correlational analyses in this study demonstrated a significant relationship between foreign language enjoyment, boredom, self-regulated strategies and perceived effectiveness. Students’ FLE was positively related to SRL strategies (rs = 0.55–0.61, ps < 0.01) and PE (*r* = 0.59, *p* < 0.01) in LMOOC learning. This suggested that students who experience more enjoyment in learning LMOOC are better at using SRL strategies and perceived more effectiveness in their LMOOC learning. This was aligned with findings in previous studies that enjoyment was a significant predictor of SRL strategies (Mega et al., 2014; You and Kang, 2014; An et al., 2021) and FLE influenced both their perceived and actual English achievement positively and significantly (Li, 2020; Li and Han, 2022). On the contrary, FLB showed a negative correlation with FLE, SRL strategies and perceived effectiveness. The relationship between enjoyment and boredom has received wide attention because both of them are achievement emotions focusing on ongoing activities but differ in value and activation. Enjoyment is activated and positive while boredom is deactivated and negative (Pekrun and Stephens, 2010). A number of studies have confirmed the negative relationship between FLE and FLB in foreign language learning contexts (Dewaele et al., 2018; Li et al., 2020). The negative relationship between boredom and SRL strategies has been found by Pekrun et al.’s (2010) and the result was also supported in online learning (Cho and Heron, 2015). The negative correlation between boredom and perceived effectiveness was consistent with Li and Han (2022) which portrayed that both boredom and anxiety were predictors of perceived achievement of online language learning. Since boredom was negatively related to SRL strategies and perceived effectiveness in LMOOCs study, more measures should be implemented to reduce students’ boredom to promote their learning strategies and achievement in online language learning contexts.

The second research question reveals the contribution of different SRL strategies to perceived effectiveness in LMOOC learning. The results showed that SRL strategies had a statistically significant effect on perceived effectiveness in our study. Researchers have widely acknowledged that self-regulated learners tend to have better language learning outcomes in online language learning (Şahin Kizil and Savran, 2018; Teng and Zhang, 2020; Bai and Wang, 2023). The three-factor structure of SRL was confirmed in our study, namely learning strategy (LS), time management (TM), and goal setting (GS). This study reported that TM was the most frequently used SRL strategy compared with LS and GS. This was in accord with previous studies which reported that time management is a critical factor in influencing student achievement in online learning (Ahmad Uzir et al., 2020). According to the multiple linear regression and Pearson correlation analysis, the three strategies were all significantly positively associated with perceived effectiveness. Time management was the most influential strategy to increase learning effectiveness among all three strategies. In MOOCs learning, GM has been found to be a significant predictor of learners’ perceived effectiveness together with meta-cognitive and environmental structuring strategies (Lee et al., 2020). This may result from the special features of MOOCs in which watching online videos takes up a large part of learning. Given the fact that students had a moderate level of SRL strategies in learning the LMOOC, there is an urgent need to improve students’ SRL strategies in LMOOCs learning. The utilization of SRL strategies should be given extra attention and it is recommended to raise students’ awareness to employ more SRL strategies while teaching foreign language in MOOCs, such as metacognitive and critical thinking strategies.

The third research question concentrates on the mediating role played by SRL strategies between students’ enjoyment, boredom and perceived effectiveness in LMOOC learning. In other words, the study intends to explore how enjoyment and boredom influence perceived effectiveness through their effect on SRL strategies. In our study, SRL strategies partially mediated the positive effects of FLE and completely mediated the negative effects of FLB on perceived effectiveness. Mega et al. (2014) confirmed that positive emotions can affect academic achievement when they are mediated by self-regulated learning. The positive relationship between FLE and SRL and the negative relationship between FLB and SRL were in line with the correlations we presented in the discussion of research question one. However, when including all variables in SEM, FLE is still significantly related to PE but the direct effect of FLB on PE was not significant. Our result was aligned with the research finding of Li and Han (2022) that boredom was not significantly related with students’ learning outcomes in the online English classroom and partly confirmed Pekrun et al.’s (2009) findings about the negative relationship between boredom and undergraduates’ performance in traditional offline courses. It may be interpreted by self-paced learning of the LMOOC in our study which provided learners freedom of self-regulated learning and rearrangement of learning activities, which helped to wear down the boredom that they encounter. In this way, learners’ perceived effectiveness is not impaired by foreign language boredom directly. The effects of boredom in LMOOCs learning are so intriguing that deserve more attention in future research. In this study, the mediating role of SRL strategies was also confirmed. The general mediating role of SRL strategies calls for teachers’ attention to consciously cultivate students’ ability to use SRL strategies while learning LMOOCs. Among the three SRL strategies in this study, time management is the only strategy that has significant mediating effects between FLE and PE. For the result of FLB, three SRL strategies were all significant mediators between FLB and PE and TM remained to be the greatest mediating effect among all the strategies. It is assumed that online learning contexts support flexible time management practices since many activities can be completed independently in an asynchronous context, at students’ own paces (Gomez et al., 2010).

## 6. Limitations and future research suggestions

Our study extends the research literature on the relationship between enjoyment, boredom, SRL strategies and academic performance to LMOOCs, laying a foundation for further investigation. However, some limitations still exist in our study. First, our research relies on self-report questionnaires to collect data. Whereas self-report seems to be the optimal method available for assessing achievement emotions (Zeidner, 1998), more advanced methods and new technologies, such as neuroimaging, and facial recognition can be utilized to minimize participants’ subjectiveness. Second, the small sample size of this study may limit the scope of empirical research. In future studies, appropriately expanding the sample size may improve the accuracy of the data and provide more valid statistical findings. Third, as positive psychologists in SLA pointed out (MacIntyre and Gregersen, 2012; Dewaele and Li, 2021), a more holistic view on diverse emotions should be embraced in future research. Other positive emotions such as hope, pride, curiosity, and excitement can also be investigated in the emergent field of LMOOCs. Finally, future research can broaden the current three-factor SRL strategies to explore more categories of SRL strategies in LMOOCs learning. Besides, self-regulated learning is a cyclical process consisting of three phases: forethought, performance, and self-reflective (Zimmerman and Moylan, 2009). The cross-sectional design could only reveal the relationship among variables at a specific time. To better investigate the dynamic relations among those variables, longitudinal research can be constructed to collect data at different stages of self-regulated learning in LMOOCs.

## 7. Conclusion

The present study investigated the relationship between foreign language enjoyment, boredom, SRL strategies, and perceived effectiveness. The mediating effects of SRL strategies between two academic emotions and perceived effectiveness were examined. The results showed that LMOOCs learners perceived a high level of enjoyment, a moderate level of boredom, and moderate levels of SRL strategies and perceived effectiveness. There were significant correlations among all the variables. FLE positively correlated with SRL and PE, while FLB negatively correlated with them. Three SRL strategies were confirmed and the predictors of PE in LMOOCs and TM played the largest predictive role. As for the third research question, the mediating effects of SRL between FLE, FLB, and PE was found. FLE was partially mediated while FLB was fully mediated. Among all SRL strategies, time management was the most effective strategy in mediating the positive effects of FLE and the negative effects of FLB on perceived effectiveness. These findings not only identified the critical role of SRL strategies in affecting students’ emotions and perceived effectiveness in LMOOC learning but also provided empirical evidence for the importance of studying students’ academic emotions in online language learning contexts.

The study provides important theoretical and practical implications for foreign language researchers and educators. Theoretically, it enriches the control-value theory of achievement emotions by broadening the study of FLE and ELB to the scope of asynchronous LMOOCs learning. This study contributes to the existing literature on LMOOCs by exploring the relationships between emotions, SRL strategies and perceived effectiveness in this emergent online language learning environment. The results of this study provides a deeper theoretical understanding of learners’ psychological mechanism in LMOOCs learning. Practically, the empirical evidence in this study highlights the crucial role played by SRL strategies in LMOOCs learning. Teachers should take more effective measures to guide students to develop effective SRL strategies for LMOOCs learning. More SRL strategy training or even small activities will encourage self-regulation in online language learning (Li, 2019). Finally, for learners, building emotional connections with LMOOCs and raising awareness of developing SRL strategies will improve their academic performance in LMOOCs learning.

## Data availability statement

The raw data supporting the conclusions of this article will be made available by the authors, without undue reservation.

## Ethics statement

Ethical review and approval was not required for the study on human participants in accordance with the local legislation and institutional requirements. The patients/participants provided their written informed consent to participate in this study.

## Author contributions

RL: conceptualization, investigation, data collection, writing and editing, and project administration. YW: data analysis, methodology, validation, and writing and editing. All authors contributed to the article and approved the submitted version.

## Funding

This research was funded by the Chinese National Social Science Fund Project “Learning Effectiveness and Quality Evaluation of Chinese Universities’ Language MOOCs” (19CYY049).

## Conflict of interest

The authors declare that the research was conducted in the absence of any commercial or financial relationships that could be construed as a potential conflict of interest.

## Publisher’s note

All claims expressed in this article are solely those of the authors and do not necessarily represent those of their affiliated organizations, or those of the publisher, the editors and the reviewers. Any product that may be evaluated in this article, or claim that may be made by its manufacturer, is not guaranteed or endorsed by the publisher.

## Supplementary material

The Supplementary Material for this article can be found online at: https://www.frontiersin.org/articles/10.3389/fpsyg.2023.1145773/full#supplementary-material
