# Supplementary material for: Enjoyment, boredom, and perceived effectiveness of learners in language MOOCs: the mediating effect of self-regulated learning
Source: Front Psychol. 2023 Jun 15;14:1145773. doi: 10.3389/fpsyg.2023.1145773 (PMC10311251; doi:10.3389/fpsyg.2023.1145773)
Supplement: Supplementary file 1 [file Table_1.docx]

**Appendix 1**

**Foreign Language Enjoyment (Li et al., 2018)**

1. I enjoy learning this LMOOC.
2. I feel proud of my accomplishments of all tasks and exams.
3. I’m excited when I learn something new in this LMOOC.
4. I have great passion for MOOC learning.
5. I prefer to learn online compared with offline learning.
6. I’m happy that this LMOOC provides diverse learning resources and a new learning mode.

**Foreign Language Boredom (Li et al., 2021)**

1. The English class bores me.
2. I start yawning and my mind begins to wander in English class because I’m so bored.
3. So many similar types of exercises in the MOOC make me lose interest.
4. The tasks are often too difficult or too easy, and I don’t want to do them.
5. Teachers in this LMOOC speak slowly and their language is not vivid, which really bore me.
6. No one answer my questions or interact with me in MOOC discussion forum
7. Teachers in the LMOOC can not give me timely response and I feel like I have learned nothing, which makes me bored.

**Online self-regulated learning (Fung et al., 2018)**

1. I try to take more thorough notes for my online courses because notes are even more important for learning online than in a regular classroom.
2. I ask myself some questions while learning this LMOOC.
3. I share my problems with my classmates online, so we know what we are struggling with and how to solve our problems.
4. I find a comfortable place to study this LMOOC.
5. I know where I can study most efficiently for online courses.
6. I contact my teacher via social media so that I can consult with him or her when I need help.
7. I read aloud instructional materials posted online to fight against distractions.
8. I summarize my learning in this LMOOC to examine my understanding of what I have learned.
9. I can guarantee weekly study time for learning this LMOOC.
10. I choose a time with few distractions for studying for this LMOOC.
11. I allocate extra studying time for my LMOOC learning because I know it is time-demanding.
12. I set short-term (daily or weekly) goals as well as long-term goals (monthly or for the semester) in learning this LMOOC.
13. I don’t compromise the quality of my work in this LMOOC because it is an online course.
14. I prepare my questions before joining in the MOOC discussion forum.
15. I arrange my learning of each chapter of this LMOOC according to my interests.
16. Although we don’t have to attend daily classes, I still try to distribute my studying time of this LMOOC evenly across days.
17. I find someone who is knowledgeable in course content of this LMOOC so that I can consult with him or her when I need help.
18. I communicate with classmates to know how is my learning performance in this LMOOC.

**Perceived effectiveness (Shen & Wu, 2020)**

1. I believe I can perform well in self-paced learning of this LMOOC.
2. I am confident that I can learn this LMOOC without the presence of an instructor to assist me.
3. Although MOOC learning is different from offline learning, I believe that I can learn more efficiently.
4. My English language skills, such as speaking, listening, improved after learning this LMOOC.
5. I think my ability to search and use online learning resources to assist learning has improved in my LMOOC learning.
6. This LMOOC broadens my cultural horizon.
